# Supplementary material for: Serum IgG titers to periodontal pathogens predict 3-month outcome in ischemic stroke patients
Source: PLoS One. 2020 Aug 6;15(8):e0237185. doi: 10.1371/journal.pone.0237185 (PMC7410289; doi:10.1371/journal.pone.0237185)
Supplement: S1 Table — (DOCX) [file pone.0237185.s002.docx]

| **Factors** | **Odds ratio** | **95% CI** | **p-value** |
| --- | --- | --- | --- |
| Age | 1.04 | 1.02-1.07 | 0.002 |
| Sex (female) | 1.21 | 0.59-2.47 | 0.60 |
| Current smoker | 1.29 | 0.63-2.66 | 0.49 |
| Habitual drinker | 0.45 | 0.23-0.87 | 0.024 |
| NIHSS score on admission | 1.22 | 1.16-1.29 | <0.001 |
| Cardioembloism | 1.41 | 0.67-2.89 | 0.36 |
| Serum CRP | 1.21 | 1.03-1.42 | 0.022 |
| *Prevotella intermedia* | 1.22 | 0.41-3.38 | 0.71 |
| *Fusobacterium* *nucleatum* | 7.64 | 3.54-16.91 | <0.001 |
| *Treponema denticola* | 0.86 | 0.36-1.97 | 0.73 |
| *Campylobacter rectus* | 1.69 | 0.68-4.06 | 0.25 |

CI, confidence interval; NIHSS, National Institutes of Health Stroke scale; IQR, interquartile range, CRP: C-reactive protein
