# Supplementary material for: Serum IgG titers to periodontal pathogens predict 3-month outcome in ischemic stroke patients
Source: PLoS One. 2020 Aug 6;15(8):e0237185. doi: 10.1371/journal.pone.0237185 (PMC7410289; doi:10.1371/journal.pone.0237185)
Supplement: Attachment — Submitted filename: Response to Reviewers.docx [file pone.0237185.s003.docx]

Jun 24, 2020

Dear Kazunori Toyoda, MD, PhD

We are most grateful to you and the reviewers for the critical comments and useful suggestions that have helped us to improve our paper considerably. As indicated in the responses as follow, we have taken all these comments and suggestions into account in the revised version of our paper.

Response to Reviewer #1

We wish to express our appreciation to the Reviewer for his or her insightful comments, which have helped us to improve the manuscript significantly.

1. **The same group published a paper showing that serum IgG antibody titer to Fusobacterium nucleatum is associated with unfavorable outcome after ischemic and hemorrhagic stroke (n=534). The main result seems to be similar although the current manuscript analyzed data only from ischemic stroke (n=445). The authors should clarify that this manuscript does not overlap substantially with the paper by Nishi et al. in Clin Exp Immunol (reference No. 13). Otherwise, the current manuscript may be subject to duplicate publication.**

Because the pathophysiology is different between ischemic stroke and hemorrhagic stroke, we believe that each characteristic becomes much clear by analyzing separately. In our study, numbers of positive for periodontal pathogens are independent predictors of unfavorable outcome in only ischemic stroke patients, not in hemorrhagic stroke patients. There is a possibility that an interaction of multiple periodontal pathogens may have negative effects on only acute infarction. On the other hand, IgG titers to *A. actinomycetemcomitans*, that do not impact on ischemic stroke patients at all, are an independent factor for predicting cerebral hemorrhage growth in hemorrhage stroke patients. In this way, IgG titers to periodontal pathogens indicate difference impacts between ischemic stroke patients and hemorrhagic stroke patients. Therefore, we think that the analysis which focuses on ischemic stroke patients has a major significance for an elucidation of relationship between periodontitis and stroke.

We have put this information in page 18-19, line 246-258.

2. **The authors suggest that Fusobacterium nucleatum elicits host responses and expanded the damage in the ischemic penumbra during the acute phase of ischemic stroke. This is an overstatement. The inflammatory response can elicit systemic responses not restricted to the ischemic penumbra as shown in an experimental study. The authors should cite the following paper (O’Boyle et al. Int J Stroke 2020) and discuss the mechanism in details.**

O’Boyle *et al*. showed that despite elevated systemic inflammation in periodontitis, oral inflammatory disease does not impact acute stroke pathology in terms of severity, determined primarily by infarct volume. However, the pathogen of periodontitis is only *P. gingivalis* in their study. In our study, IgG titers to *P. gingivalis* did not have an effect on outcomes in ischemic stroke patients. On the other hand, *F.nucleatum* is reported to be able to pass through the blood-brain barrier and has been found to be causative of brain abscesses in some case studies, while it also has abilities to adhere to and invade host vascular endothelial cells via FadA adhesion molecules, as FadA binds to vascular endothelial-cadherin on the cell surface, which triggers breakdown of endothelial cell-to-cell junctions. Following passage through the blood-brain-barrier, *F.nucleatum* organisms attack vascular endothelial cells in blood vessels in the brain, which can induce endothelial permeability via loosened cell junctions. We hypothesize that these mechanisms induce expanding the damage in the ischemic penumbra during the acute phase of ischemic stroke. This result suggests that the inflammatory response differs depending on the pathogen of periodontitis.

We have put this information in page 19-20, line 269-284.

3. **As discussed by the authors, the biggest limitation of this study is lack of oral assessment. There still remains a possibility that poor stroke outcome is associated with general oral (periodontal) health but not associated with specific periodontal pathogens if the elevated titers reflect periodontal health. The authors have to incorporate their own or published data that can deny the possibility.**

It is true that we did not evaluate the oral conditions such as tooth loss and grade of periodontal disease in most patients. We cannot eliminate the possibility that these factors can influence the outcome in ischemic stroke patients. However, several reports have shown that IgG levels against periodontal pathogens are more accurate measures of periodontal infection and its severity. Takeuchi Y et al. showed that higher anti-P. gingivalis IgG levels were found in the periodontitis group compared with the healthy control group. Pussinen PJ et al. found that ELISA is suitable for measuring antibodies to periodontal pathogens in large epidemiological studies in order to evaluate the role of periodontitis as a risk factor for other diseases. Kudo C et al. revealed that IgG titers of periodontitis patients were significantly higher than those of healthy controls, especially in those with sites of probing depth over 4 mm. In this way, IgG titers to periodontal pathogens are established as a surrogate marker of periodontal health. Moreover, we assessed the tooth status of 85 patients. The tooth status was evaluated by a dentist within 7 day after admission. The median numbers of remaining tooth were 23 (IQR 14-26), and treated tooth was 9 (IQR 4-14). Patients with unfavorable outcome were significantly lower numbers of remaining tooth than patients with favorable outcome. (20 [IQR 10-25] vs. 24 [IQR 16-27], p=0.03). We found that a positive correlation between the numbers of tooth loss and the numbers of positive for periodontal pathogens. Therefore, we believe that this limitation does not have a substantial influence on our results.

We have put this information in page 21-22, line 293-307.

4. **In the Abstract, the name of bacteria should be fully spelled out.**

We fully spelled out the name of periodontal pathogens in the abstract section.

Response to Reviewer #2

We wish to express our appreciation to the Reviewer for his or her insightful comments, which have helped us to improve the manuscript significantly.

1. **PD linked with socioeconomic status and access to dental care. Inclusion of the variables in model.**

In this study, we did not evaluate the socioeconomic status and access to dental care in each patient. We cannot eliminate the possibility that these factors can influence the outcome in ischemic stroke patients. However, several reports have shown that IgG levels against periodontal pathogens are more accurate measures of periodontal infection and its severity. Takeuchi Y et al. showed that higher anti-P. gingivalis IgG levels were found in the periodontitis group compared with the healthy control group. Pussinen PJ et al. found that ELISA is suitable for measuring antibodies to periodontal pathogens in large epidemiological studies in order to evaluate the role of periodontitis as a risk factor for other diseases. Kudo C et al. revealed that IgG titers of periodontitis patients were significantly higher than those of healthy controls, especially in those with sites of probing depth over 4 mm. In this way, IgG titers to periodontal pathogens are established as a surrogate marker of periodontal health. Moreover, we assessed the tooth status of 85 patients. The tooth status was evaluated by a dentist within 7 day after admission. The median numbers of remaining tooth were 23 (IQR 14-26), and treated tooth was 9 (IQR 4-14). Patients with unfavorable outcome were significantly lower numbers of remaining tooth than patients with favorable outcome. (20 [IQR 10-25] vs. 24 [IQR 16-27], p=0.03). We found that a positive correlation between the numbers of tooth loss and the numbers of positive for periodontal pathogens. Therefore, we believe that this limitation does not have a substantial influence on our results.

We have put this information in page 21-22, line 293-307.

2. **Adjustment for multiple comparisons.**

We appreciate for your suggestion. However, to our understand, we made no multiple comparison in our article. And, it is not appropriate to make multiple comparisons in this study, we believe. If you, further, suggest to make multiple comparisons and its adjustment, please let us know what kind of analysis you want us to add.

3. **Some evidence to the hypothesis that PD may worsen ischemic penumbra.**

O’Boyle *et al*. showed that despite elevated systemic inflammation in periodontitis, oral inflammatory disease does not impact acute stroke pathology in terms of severity, determined primarily by infarct volume. However, the pathogen of periodontitis is only *P. gingivalis* in their study. In our study, IgG titers to *P. gingivalis* did not have an effect on outcomes in ischemic stroke patients. On the other hand, *F.nucleatum* is reported to be able to pass through the blood-brain barrier and has been found to be causative of brain abscesses in some case studies, while it also has abilities to adhere to and invade host vascular endothelial cells via FadA adhesion molecules, as FadA binds to vascular endothelial-cadherin on the cell surface, which triggers breakdown of endothelial cell-to-cell junctions. Following passage through the blood-brain-barrier, *F.nucleatum* organisms attack vascular endothelial cells in blood vessels in the brain, which can induce endothelial permeability via loosened cell junctions. We hypothesize that these mechanisms induce expanding the damage in the ischemic penumbra during the acute phase of ischemic stroke. This result suggests that the inflammatory response differs depending on the pathogen of periodontitis.

We have put this information in page 19-20, line 269-284.

4. **Animal data on F nucleatum, why other pathogens were not predictors of unfavorable outcome.**

*F.nucleatum* is reported to be able to pass through the blood-brain barrier and has been found to be causative of brain abscesses in some case studies, while it also has abilities to adhere to and invade host vascular endothelial cells via FadA adhesion molecules, as FadA binds to vascular endothelial-cadherin on the cell surface, which triggers breakdown of endothelial cell-to-cell junctions. Following passage through the blood-brain-barrier, *F.nucleatum* organisms attack vascular endothelial cells in blood vessels in the brain, which can induce endothelial permeability via loosened cell junctions. We hypothesize that these mechanisms induce expanding the damage in the ischemic penumbra during the acute phase of ischemic stroke. This result suggests that the inflammatory response differs depending on the pathogen of periodontitis.

We have put this information in page 19-20, line 274-284.

5. **Basic information on tooth status would be useful as well.**

In this study, we assessed the tooth status of 85 patients. The tooth status was evaluated by a dentist within 7 day after admission. The median numbers of remaining tooth were 23 (IQR 14-26), and treated tooth was 9 (IQR 4-14). Patients with unfavorable outcome were significantly lower numbers of remaining tooth than patients with favorable outcome. (20 [IQR 10-25] vs. 24 [IQR 16-27], p=0.03). We found that a positive correlation between the numbers of tooth loss and the numbers of positive for periodontal pathogens.

We have put this information in page 21-22, line 301-307.
