# Supplementary material for: Serum IgG titers to periodontal pathogens predict 3-month outcome in ischemic stroke patients
Source: PLoS One. 2020 Aug 6;15(8):e0237185. doi: 10.1371/journal.pone.0237185 (PMC7410289; doi:10.1371/journal.pone.0237185)
Supplement: Attachment — Submitted filename: Response to Reviewers.docx [file pone.0237185.s004.docx]

Jul 20, 2020

Dear Kazunori Toyoda, MD, PhD

We are most grateful to you and the reviewers for the critical comments and useful suggestions that have helped us to improve our paper considerably. As indicated in the responses as follow, we have taken all these comments and suggestions into account in the revised version of our paper.

Response to Reviewer #1

We wish to express our appreciation to the Reviewer for his or her insightful comments, which have helped us to improve the manuscript significantly.

1. **The authors have added some comments on the difference between ischemic and hemorrhagic stroke in the discussion (page 18). Cite the reference 13 in the discussion, and discuss more concretely by showing the results obtained in the reference 13 and the current paper.**

Nishi et al. previously reported that IgG titers to a specific periodontal pathogen (*F. nucleatum*) were associated with unfavorable outcome after all subtypes of stroke. On the other hand, we performed the comprehensive analysis of relationship between IgG titers to whole periodontal pathogens and acute ischemic stroke. To clarify this difference, we deleted the description about relationship between IgG titers to *F. nucleatum* and outcome in Abstract section and result section (table 4 [model 1]). Additionally, we revised the fourth paragraph of Discussion section as follow underlined (page 18-19, line 247-264)

In the present study, numbers of IgG antibodies positive for periodontal pathogens are independent predictors of unfavorable outcome in only ischemic stroke patients, not in hemorrhagic stroke patients. There is a possibility that an interaction between multiple periodontal pathogens may have negative effects on acute infarction. However, whether periodontal disease can modify acute ischemic brain damage is not fully understood. O’Boyle et al. showed that despite elevated systemic inflammation in periodontitis, oral inflammatory disease does not impact acute stroke pathology in terms of severity, determined primarily by infarct volume [32]. On the other hand, periodontal pathogens themselves are highly invasive; *P. gingivalis* can compromise and cross the blood brain barrier into the brain [33], and *Treponema spp.* have been speculated to enter the brain directly via peripheral trigeminal nerves [34]. *F.nucleatum* is also reported to be able to pass through the blood-brain barrier and has been found to be causative of brain abscesses in some case studies [35, 36]. Multiple periodontal pathogens infections are considered to cause higher-grade systemic inflammation. Several reports showed that high-grade systemic inflammation is deleterious in the context of ischemic stroke [37-39]. We speculate that this is a part of the reasons why numbers of IgG antibodies positive for periodontal pathogens are independent predictors of unfavorable outcome in ischemic stroke patients. Further studies are needed to clarify this point.

2. **Although the authors elaborated on the mechanism by which F. nucleatum attack the blood vessels. They hypothesized the mechanism as stated in the Discussion (page 20) but it was really an overstatement as this reviewer suggested earlier. Why can the authors say the bacteria expand the damage in the penumbra. In particular, why can the authors pinpoint the site of damage in the penumbra? Without any experimental evidence, one cannot overstate the underlying mechanisms.**

As the reviewer indicated, the speculation that *F. nucleatum* elicits host responses and expanded the damage in the ischemic penumbra during the acute phase of ischemic stroke is an overstatement. We revised the fourth paragraph of Discussion section as follow underlined (page 18-19, line 247-264)

In the present study, numbers of IgG antibodies positive for periodontal pathogens are independent predictors of unfavorable outcome in only ischemic stroke patients, not in hemorrhagic stroke patients. There is a possibility that an interaction between multiple periodontal pathogens may have negative effects on acute infarction. However, whether periodontal disease can modify acute ischemic brain damage is not fully understood. O’Boyle et al. showed that despite elevated systemic inflammation in periodontitis, oral inflammatory disease does not impact acute stroke pathology in terms of severity, determined primarily by infarct volume [32]. On the other hand, periodontal pathogens themselves are highly invasive; *P. gingivalis* can compromise and cross the blood brain barrier into the brain [33], and *Treponema spp*. have been speculated to enter the brain directly via peripheral trigeminal nerves [34]. *F.nucleatum* is also reported to be able to pass through the blood-brain barrier and has been found to be causative of brain abscesses in some case studies [35, 36]. Multiple periodontal pathogens infections are considered to cause higher-grade systemic inflammation. Several reports showed that high-grade systemic inflammation is deleterious in the context of ischemic stroke [37-39]. We speculate that this is a part of the reasons why numbers of IgG antibodies positive for periodontal pathogens are independent predictors of unfavorable outcome in ischemic stroke patients. Further studies are needed to clarify this point.

3. **The added texts should be thoroughly checked by a native English speaker.**

As the reviewer indicated, the added texts were checked by a native English speaker.
